# Application of Laparoscopic Radical Resection for Type III and IV Hilar Cholangiocarcinoma Treatment

**DOI:** 10.1155/2020/1506275

**Published:** 2020-02-25

**Authors:** Sulai Liu, Xinyu Liu, Xuepeng Li, Ou Li, Weimin Yi, Junaid Khan, Pingzhou Yang, Chao Guo, Chuang Peng, Bo Jiang

**Affiliations:** ^1^Department of Hepatobiliary Surgery, Hunan Provincial People's Hospital/The First Affiliated Hospital of Hunan Normal University, Changsha, 410005 Hunan Province, China; ^2^Biliary Disease Research Laboratory of Hunan Provincial People's Hospital, Key Laboratory of Hunan Normal University, Changsha, 410005 Hunan Province, China; ^3^Clinical Medical Technology Research Center of Hunan Provincial for Biliary Disease Prevention and Treatment, Changsha, 410005 Hunan Province, China

## Abstract

**Background:**

This study is aimed at investigating the feasibility and safety of the laparoscopic radical resection for treating type III and IV hilar cholangiocarcinoma (III/IV Hilar C).

**Methods:**

Six patients with III/IV Hilar C were enrolled in our hospital. All patients underwent total laparoscopic surgery, including basic surgery (laparoscopic gallbladder, hilar bile duct, and common bile duct resection and hepatoduodenal ligament lymph node dissection) combined with left hepatic and caudate lobe resection/portal resection. The tumor size, operation time, intraoperative blood loss, and postoperative complications were observed. The follow-up of the patients after discharge was recorded.

**Results:**

Surgery was successfully completed in 6 patients. We found that the tumor size of 6 patients ranged from 1.5 to 3.6 cm, with 4 lymph nodes. The operation time was 540-660 minutes, and the blood loss was 300-500 ml. One patient developed bile leakage after surgery, healed within 2 weeks after drainage. The postoperative hospital stay was 16 (13-24) days. There were 4 cases of negative bile duct margin tumor, 1 case was positive, and 1 case was not reported. All 6 patients were discharged smoothly without perioperative death. Regular examinations were conducted every 3 months after discharge, and the median duration was 7 months. Only 1 patient had a marginal dysplasia, and 5 patients had no obvious signs of recurrence.

**Conclusions:**

Application of laparoscopic radical resection for III/IV Hilar C is safe and feasible and has good short-term efficacy with adequate preoperative evaluation, appropriate case selection, and precise operative strategy.

## 1. Background

Cholangiocarcinoma (CC) accounts for 10–20% of primary liver tumors and is the second most common primary hepatic cancer. In particular, 50–67% of CC cases are hilar cholangiocarcinoma (Hilar C); Hilar C is a malignant tumor with poor 5-year survival rate [1]. Moreover, Hilar C can be distinguished in four different types according to the Bismuth-Corlette classification, based on the perihilar longitudinal extension [1]. It was mainly found in the common hepatic duct, left and right hepatic ducts, and confluent bile duct mucosa. And most of the patients are sporadic without any identified susceptibility genes [2, 3]. It is believed that the risk factors of Hilar C include primary sclerosing cholangitis, liver parasitic infections, and cholelithiasis [2, 3].

Nowadays, surgical operation is the only way to improve the long-term survival rate and life quality of patients [4, 5]. Laparoscopic technology has been widely used in almost all of abdominal surgeries and achieved good clinical treatment results [6–8]. However, because of the deep location, small space, complex anatomical structures, and abundant peripheral blood vessels, surgical management of Hilar C is one of the most challenging operations for hepatobiliary surgeons [9]. Accumulation of experience and advanced techniques are needed for it to be recommended. Here, we reported 6 patients who underwent laparoscopic radical resection of type III and IV Hilar C from April 2015 to October 2018. Under adequate preoperative assessment, reasonable case selection, and rigorous surgical planning, we attempted to perform basic procedures (laparoscopic gallbladder, hilar bile duct, and common bile duct resection and hepatoduodenal ligament lymph node dissection). On the basis of considering the involvement of adjacent tissues and organs in the operation, we hope to improve the cure rate of type III and IV hilar cholangiocarcinoma.

## 2. Methods

### 2.1. Patients

All studies were approved by the Medical Ethics Committee of our hospital (RS2016909900). Prior to the inclusion in the study, 6 patients who underwent laparoscopic III and IV hilar cholangiocarcinoma were informed and provided written consent. Six patients including 4 males and 2 females, age 35-75 years, with a median age of 53 years, received laparoscopic radical resection from April 2015 to October 2018. There were 1 case of Bismuth IIIA type, 2 cases of Bismuth type IIIB, and 3 cases of Bismuth type IV. There were 1 case of TNM stage and 5 cases of T2N0M0. There were 1 case of stage IIIB in R stage and 5 cases of stage II. The information of patients is shown in Table 1 and [Supplementary-material supplementary-material-1].

All 6 patients in this study were subjected to rigorous selection and adequate preoperative assessment. Our clinical experience begins with the evaluation of tumor resectability.

- Patients underwent liver-enhanced CT or enhanced MRI before surgery, based on imaging findings, to understand the extent of tumor invasion and its relationship with adjacent tissues and to determine whether there is an invasion of the portal vein or hepatic artery and its branches
- There are conditional three-dimensional reconstruction and virtual hepatectomy and even 3D model printing
- There is a stereoscopic representation of the extent of the tumor and its influence on the liver parenchyma and intrahepatic bile duct
- There is an anatomical positional relationship between the tumor and the surrounding blood vessels, in order to establish the residual liver volume which is greater than 40% of total liver volume and R0 resection for the purpose, followed by surgical safety assessment
- In addition to preoperative routine examinations and tests, attention should be paid to the assessment of nutrition, physical fitness, respiration, and control of hypertension and diabetes
- In patients with chronic hepatitis B, regardless of their HBV DNA copy number, antiviral therapy was performed from the time the surgery was decided. For patients with severe obstructive jaundice, PTCD may be reduced. If jaundice does not decrease or increase after PTCD, it is temporary. There is no need to consider radical surgery

### 2.2. Surgical Preparation

Total laparoscopic surgery was successfully completed in 6 patients. After general anesthesia, the patient was lying flat with his head up and slightly turned to left side position. The surgeon was on the left of the patient and dissected lymph node around the liver and porta hepatis and performed portojejunal anastomosis. The doctor on the right side performed the removal of the hepatic and caudal lobe. The assistant stood on the opposite side of the surgeon, and the helper stood on the left side of the patient. The port positions were performed as a five-hole method. A 4-5 cm incision was made in the middle of the patient's abdomen to bring the specimen into the abdomen. Pneumoperitoneum was established by 10 mm longitudinal incision for a puncture on the umbilicus and maintained at 10~16 mmHg. After that, 10 mm cone sheath was inserted into the enterocoelia to examine the injury of internal organs. A 12 mm main working cannula was placed into the costal margin under the middle of the left clavicle. Meanwhile, a 5 mm auxiliary operation cannula was placed in the mid of the navel and xiphoid. Then, a 12 mm main liver operation cannula was inserted 2 cm below the costal margin under the middle of the right clavicle. And a 5 mm auxiliary cannula was placed 2 cm below the costal margin under the right anterior axillary line.

### 2.3. Perihepatic and Lymph Node Dissection

After exploration, the ligamentum teres hepatis was dissected, followed by the secondary porta of the liver and the perihepatic stripping. Then the common hepatic artery was elevated after the removal of 8^th^ and 9^th^ groups of lymph nodes. Subsequently, we went through the skeletonized proper hepatic artery, gastroduodenal artery, and common bile duct in the upper edge of the pancreas crosses. After hepatoduodenal ligament and gallbladder stripping, the hepatic portal was dissected from the bottom to the top. Thereafter, we ligated the left and right hepatic vessel bifurcation, followed by cutting off the lateral hepatic artery, portal vein, and short vein. Finally, the third porta of the liver was dissected.

### 2.4. Liver Resection

**Figure 1:**
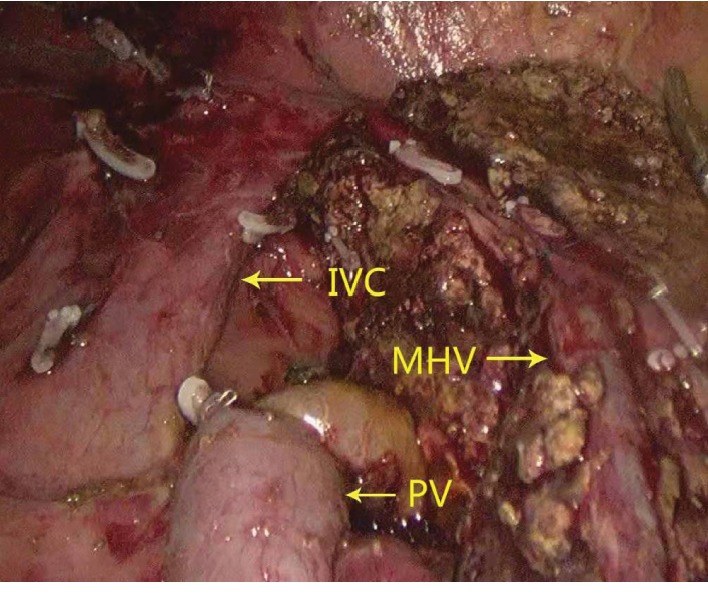
Left vision of postoperative liver section.

**Figure 2:**
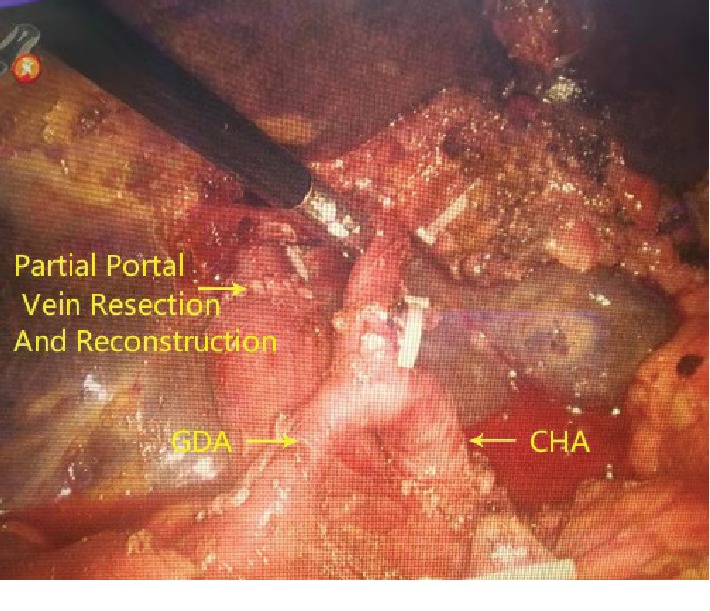
Right vision of postoperative liver section.

The central venous pressure was maintained at 3 cm water column, and the ischemic boundary line was recorded by the assistant with an electrocoagulation hook. The surgeon dissected the liver to the secondary porta with an ultrasonic small incision, disconnected the hepatic vein with a linear cutting stapler (white nail), and removed the specimen (Figures 1 and 2). During the operation, the blood vessels of 2 mm or less were coagulated, and 2 mm or more were clipped. The proper hepatic artery and portal vein were blocked by endoscopic noninvasive vascular occlusion forceps (15 + 5 mode). Along with the branch, we found and exposed the middle hepatic vein (MHV) until the secondary porta of the liver to protect the healthy lateral hepatic artery and portal vein. Subsequently, the bile duct was cut off as far as possible from the hepatic portal tumor.

### 2.5. Bile Duct Reconstruction

To achieve R0 status, multiple bile duct cuts were necessary for the specimen to be removed. Hepatic ducts were sutured and closed to form a bile duct basin. The upper jejunum was cut at a distance of 25 cm under the inferior border of the Treitz ligament with a cutter stapler. Then the hepatic duct was anastomosed with the proximal jejunum 50 cm below the distal end using a linear stapler. And the distal jejunum was lifted through the mesentery to anastomose with a bile duct. If there were a large number of tiny cuts, we could directly close the posterior wall bile duct basin. And the anterior wall could be stitched to the nearby liver tissue with a 4-0 prolene suture. To prevent the liver from being cut, we should gather the sutures after the anterior wall is stitched. Then the tube should be placed in the loop for extravasation. Or we could place the abdominal drainage tube behind the hepatic and intestinal anastomosis and near the liver section after water injection test in the 8^th^ urinary catheters.

### 2.6. Vessel Operation

**Figure 3:**
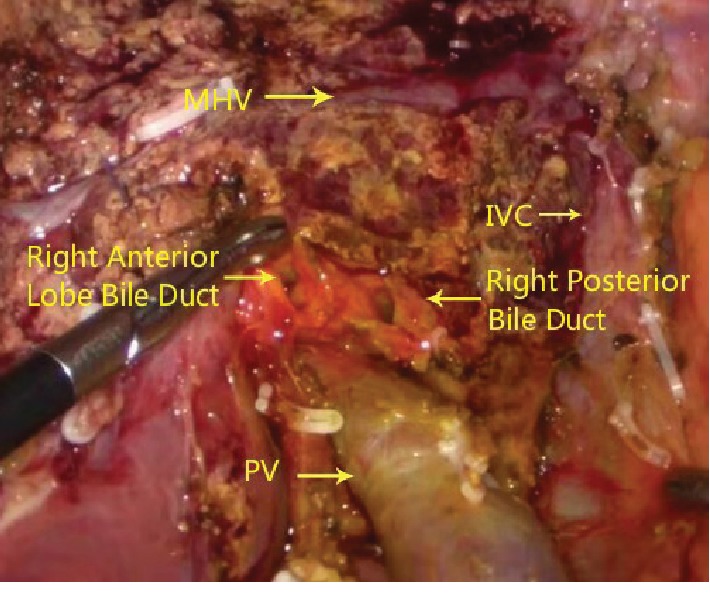
Resection and reconstruction of the portal vein.

As we know, Hilar C often associates with vascular invasion. It was necessary to perform the vascular resection and reconstruction operation, when the bifurcation of the hepatic portal vein or artery was affected. After disconnecting the hepatoduodenal ligament and cutting off the hepatic artery, the portal branch was separated until invading the site of the bifurcation. Then the end of the common bile duct was pulled up to suspend the right branch of the portal vein at the right posterior side. Subsequently, noninvasive vascular occlusion forceps were used for the portal vein and right branch of the portal vein blocking. Then affected confluence vessels were cut off and washed by heparin sodium solution after the portal vein wall was cut 2-3 mm away from the tumor invasion site. After suturing 4-5 stitches with 5-0 prolene, the suture was closed to make the posterior wall aligned (Figure 3). After operation, the residual vascular wall was about 1/4 of the circumference of the branch of the portal vein.

## 3. Results

After surgery, we observed that the tumor size of 6 patients was 1.5-3.6 cm, and there were 4 lymph nodes. The operation time was 540-660 minutes, and the blood loss was 300-500 ml. None of the patients needed blood transfusion. Only basic surgery (laparoscopic gallbladder, hilar bile duct, and common bile duct resection and hepatoduodenal ligament lymph node dissection) was combined with left hepatic, caudate lobe resection and portal resection reconstruction. In patients with postoperative bile leakage, drainage healed within 2 weeks. Only patients with basic surgery (laparoscopic gallbladder, hilar bile duct, and common bile duct resection and hepatoduodenal ligament lymph node dissection) were combined with left hepatic, caudate lobe resection and biliary anastomosis-resected marginal atypical hyperplasia. There were 4 cases of negative bile duct margin tumor, 1 case was positive, and 1 case was not reported. The patients were discharged from the hospital (16~24) days after operation, and there was no perioperative death. Six patients were collected. We collected data from multiple outpatient visits after surgery and regularly reviewed for 3 months' time with a median duration of 7 months. Six patients were still alive, 5 patients had no obvious signs of recurrence, and 1 patient lacked clinical data. All patients were able to take care of themselves (Table 2).

## 4. Discussion

Laparoscopic surgery is a newly developed minimally invasive method and an inevitable trend in the development of future surgical methods [10]. The advantage of laparoscopic radical resection for cholangiocarcinoma treatment is listed as follows:

1. With this technique, the surgery could be visible and more precise, easy, and safe
2. Because of the variable visibility angles and magnification effect, it could be easy for structural discrimination
3. The establishment of pneumoperitoneum, lower central venous pressure, and hepatic vascular occlusion combined with ultrasonic scalpel could significantly reduce the amount of blood loss during surgery
4. The application of laparoscopic radical resection could shorten the recovery time and reduce the rate of complication

Previous studies have used nonanatomical repeated laparoscopic hepatectomy for recurrent liver cancer, and this procedure is safe and feasible [11]. In laparoscopic hepatectomy, it may be an effective and feasible method to expose the vein from the trunk to the peripheral branch through the abdominal approach [12]. Single-incision laparoscopic biliary bypass for the treatment of malignant obstructive jaundice is safe and feasible, and the short-term results are satisfactory [13].

As a rare malignant tumor, surgical treatment is currently the only effective treatment for Hilar C. However, due to the complexity of the operation, the application of radical resection for Hilar C is still relatively rare [14].

Six patients with type III and IV hilar cholangiocarcinoma included in this study underwent total laparoscopic surgery. After the operation, only 1 case had postoperative bile leakage and vascular invasion, healed within 2 weeks after drainage. The other 5 patients had no complications after the operation and healed well, with no local or regional recurrence and associated complications. One case with biliary leakage healed within 2 weeks after drainage.

The summary of our clinical experience is that the location and number of the trocar are a critical step for laparoscopic radical resection. Also, the correct position of the patient, surgeon, and assistant and good surgical cooperation could make the surgery more successful and reduce bleeding, injuries, and comorbidities. To find the gap between the vessel and liver parenchyma, we should pay more attention to several important markers, such as ischemic line, hepatic vein, venous ligament fissure, posterior inferior vena cava, and border of liver fibrosis. Control of bleeding is the most important thing during a liver resection period. The easy bleeding sites include 8 ventral and interlobular veins which are close to the secondary porta, 5 main vessels, and 8 side branches which are located in the upper and lower thirds of the middle hepatic vein. During the operation, we disconnected the arteries followed by veins to reduce the risk of bleeding. In addition, we should dissect the liver carefully with the high tension, thin walls, and large number of vessels around the tumor. Bleeding should be treated carefully in the period of operation. However, these tumors are often grey-white scirrhous masses with a poor vascularization, unlike hepatocarcinoma [15]. If the portal vein is affected, portal vein resection and reconstruction are required. Taking advantage of the amplification effect and close observation of laparoscopy, we could control the needle and edge spacing in a better way. Before resection, we must determine the length of the blood vessel dissection to achieve R0 status and facilitate vessel reconstruction. Then we should separate the distal and proximal ends of the blood vessel dissection for vascular occlusion. These techniques can help us complete the surgery successfully. Though disputed, most of the researchers believe that caudate lobe dissection is necessary for Hilar C treatment [16, 17]. The caudate lobe is located between the inferior vena cava and the portal vein, surrounded with dense and important blood vessels [18, 19]. When dealing with the short hepatic vein, slight mistake might lead to tearing of the inferior vena cava and induce uncontrolled bleeding. In the utilization of laparoscopy, we could expose the caudate lobe and left portal vein completely before surgery, which could reduce the difficultly of caudate resection.

In this study, 6 patients with type III and IV hilar cholangiocarcinoma who underwent total laparoscopic hilar cholangiocarcinoma were successfully operated without serious complications and death.

## 5. Conclusions

Our preliminary clinical practice shows that type III and IV hilar cholangiocarcinoma and combined invasive portal vein resection and reconstruction of type III and IV livers are ensured under the premise of adequate preoperative assessment, reasonable case selection, and rigorous surgical planning. Portal cholangiocarcinoma is safe and feasible in laparoscopic radical surgery, and the short-term effect is good. However, its long-term efficacy, whether it can improve the survival rate of patients, still needs continued follow-up. It should not be overlooked that there are few cases in this study, and the conclusion still needs more laparoscopic surgery to treat cases of type III and IV hilar cholangiocarcinoma.

## Figures and Tables

**Table 1:** General data of 6 patients undergoing laparoscopic radical resection of hilar cholangiocarcinoma.

Number	Sex	Age (Y)	Diagnosis	Bismuth classification	Pre-op	TB (*μ*mol/l)
1	Female	35	PHCC	IIIB	—	17.6
2	Male	45	PHCC	IV	PTCD	345.1
3	Female	62	PHCC	IV	PTCD	325.9
4	Male	37	PHCC	IIIB	—	72.3
5	Male	75	PHCC	IIIA	—	94.6
6	Male	73	PHCC	IV	—	36.7

PHCC: perihilar cholangiocarcinoma; PTCD: percutaneous transhepatic cholangio-drainage; —: lacking.

**Table 2:** Six patients' operation and prognosis.

Case	Joint surgery	Operation time (min)	Amount of bleeding (ml)	Complication	Bile duct margin	Median size of tumor	Vascular invasion	Atypical hyperplasia
1	Left hepatic+caudate lobe	540	300	—	P	2.1 cm	N	N
	Resection+biliary anastomosis							
2	Left hepatic+caudate lobe	660	300	BL	No	3.6 cm	Y	N
	Resection+portal vein resection+biliary anastomosis				Report			
3	Left hepatic+caudate lobe	600	500	—	p	1.5 cm	N	N
	Resection+biliary anastomosis							
4	Left hepatic+caudate lobe	540	300	—	P	1.7 cm	N	N
	Resection+biliary anastomosis							
5	Right hepatic+caudate lobe	660	500	—	P	2.4 cm	N	N
	Resection+biliary anastomosis							
6	Left hepatic+caudate lobe	540	500	—	N	2.0 cm	N	Y
	Resection+biliary anastomosis							

Basic operation was defined as gallbladder, hilar, and common bile duct resection and lymph node dissection. BT: blood transfusion; LOS: length of stay in hospital; BD: bile ducts; BL: bile leakage; N: negative; P: positive; Y: yes; N: no.

## Data Availability

Data and materials are included in the manuscript.

## Abbreviations

Hilar C: Hilar cholangiocarcinoma
MHV: Middle hepatic vein.

## Supplementary Materials

Supplementary Materials Table S1: supplementary information of 6 patients undergoing laparoscopic radical resection of hilar cholangiocarcinoma.
