# Supplementary material for: Application of Laparoscopic Radical Resection for Type III and IV Hilar Cholangiocarcinoma Treatment
Source: Gastroenterol Res Pract. 2020 Feb 25;2020:1506275. doi: 10.1155/2020/1506275 (PMC7060852; doi:10.1155/2020/1506275)
Supplement: Supplementary Materials — Table S1: supplementary information of 6 patients undergoing laparoscopic radical resection of hilar cholangiocarcinoma. [file 1506275.f1.pdf]

Supplementary Material

Table S1 Supplementary information of 6 patients undergoing laparoscopic radical resection of hilar cholangiocarcinoma

case	Median size of tumor	TNM stage	R stage	Atypical hyperplasia	Vascular invasion	knots
1	2.1cm	T4N0M0	IIIB	N	N	4
2	3.6cm	T2N0M0	II	N	Y	4
3	1.5cm	T2N0M0	II	N	N	4
4	1.7cm	T2N0M0	II	N	N	4
5	2.4cm	T2N0M0	II	N	N	4
6	2.0cm	T2N0M0	II	Y	N	4
